# Ultrathin Co-O oxide layer-driven perpendicular magnetic anisotropy in a CoO/[Co/Pd]_m_ multilayer matrix upon annealing

**DOI:** 10.1038/srep37503

**Published:** 2016-11-25

**Authors:** Woo Seong Chung, Seung Mo Yang, Tae Whan Kim, Jin Pyo Hong

**Affiliations:** 1 Nano Quantum Electronics Lab, Department of Electronics and Computer Engineering, Hanyang University, Seoul, 133-791, South Korea; 2 Novel Functional Materials and Devices Laboratory, Research Institute for Natural Science, Department of Physics, Hanyang University, Seoul, 133-791, South Korea

## Abstract

Ferromagnetic/noble metal multilayer (ML) frames are expected to serve as reliable building blocks in a variety of perpendicular magnetic anisotropy (PMA) based spintronic devices. However, ultrathin ML matrices are highly susceptible to unintended reduction of electron spin polarization in the as-grown or annealed states and often require a large repeat number. Here, we introduce a simple approach to achieve thermally stable PMA in ultrathin [Co/Pd]_3_ MLs involving the incorporation of an ultrathin CoO capping layer. The thickness and oxygen content of the CoO layer are critical parameters to achieve enhanced PMA in ultrathin [Co/Pd]_3_/CoO MLs post-annealed up to 400 °C. An extensive analysis of structural features identified that robust PMA characteristics in [Co/Pd]_3_/CoO MLs are linked with thermally activated oxygen atom diffusion leading to structural reconfiguration upon annealing. The possible origin of the enhanced PMA in our [Co/Pd]_3_/CoO ML samples after high-temperature annealing is discussed, thereby enabling their use in future spintronic-related devices.

Spin-transfer torque magnetic random access memories (STT-MRAMs) have generated considerable interest as one of the most attractive candidates for emerging next-generation nonvolatile memory due to their extremely low power consumption, high-speed performance, and non-volatility^1^,^2^. Perpendicular magnetic tunnel junctions (*p-*MTJs) containing perpendicular magnetic anisotropy (PMA) have outstanding thermal stability (Δ = 74) and enable more efficient current switching than in-plane MTJs (*i-*MTJs)^3^,^4^,^5^. A variety of PMA materials including rare earth-transition metal (RE-TM) films, *L*1_0_-(001)-oriented Fe/Pt or Co/Pt films, and ferromagnetic metal/noble metal (FM/NM) artificial multilayer (ML) matrices like Co/Pt, Co/Pd, Fe/Pt, Fe/Pd have been employed^6^,^7^,^8^,^9^. In particular, Co-based MLs have been the focus of intense investigation because of their strong PMA characteristics, which can be tuned by selecting suitable repeat numbers (the number of times the two layers are repeated in the ML stack) and by alternating the thickness of each layer^10^. It is widely believed that a large repeat number in a [FM/NM] ML stack gives rise to strong interface PMA due to structural and orbital asymmetry, i.e. interface spin orbit coupling between FM and NM layers^10^,^11^. Meanwhile, an ultrathin ML matrix with a small repeat number (*m* = 3 in this letter) is susceptible to rapid PMA degradation induced by atomic intermixing and deterioration of the interface anisotropy in both the as-grown and annealed states^12^. Our previous work addressed the possibility of using an NiO_x_ capping layer to enhance the PMA in [Co/Pd]_3_ MLs, where a large number of oxygen atoms were inserted during sputtering to build up additional Co/Oxide interface PMA which remains stable even after high-temperature annealing^12^. The alternate possibility of incorporating a [CoO/Pd]_2_ ML buffer layer into a thick [Co/Pd]_7_ ML frame for thermally activated diffusion of oxygen atoms leading to atomic structural reconfiguration during annealing was also examined^13^. However, widespread use of ultrathin Co/Pd MLs is still limited by incomplete structural reconfiguration in the ML frame, which negatively impacts the PMA performance. As such, practical questions still remain about how oxide-based capping layers behave during annealing, and how a suitable capping layer should be made.

Substantial efforts have been directed to achieve epitaxial growth of an ordered ML matrix through control of the crystal structure and c-axis orientation, including by substrate heating, post-annealing, and/or insertion of a proper capping/buffer layer^13^,^14^. In this letter, we report a simple CoO capping approach which results in appreciable PMA in an ultrathin [Co/Pd]_3_ ML matrix after high-temperature annealing. The thickness and oxygen content of the incorporated CoO layer are critical to achieving strong PMA. Magnetic and structural characteristics were analyzed by vibrating sample magnetometer (VSM) and high-resolution X-ray diffraction (HR-XRD), respectively. In addition, atomic depth profile analyses of oxygen and cobalt species were conducted by X-ray photoemission spectroscopy (XPS). We show that the enhanced PMA in ultrathin CoO_x_-capped ML frames likely arises from atomic ML reconstruction induced by oxygen atom diffusion during high-temperature annealing.

## Results and Discussion

### Comparison of CoO capping layer and Co capping layer

**Figure 1:**
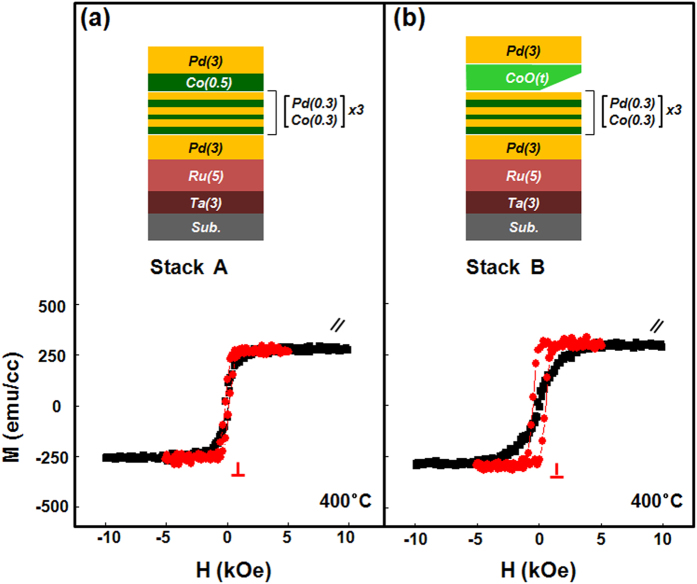
Schematic structures and in-plane (black)/out-of-plane (red) magnetic hysteresis loops of (**a**) [Co(0.3)/Pd(0.3)]_3_/Co-capping samples without oxygen insertion (Stack A) and (**b**) [Co(0.3)/Pd(0.3)]_3_/CoO-capping samples (Stack B), where the numbers in parentheses represent the thickness of the film in nanometers. Both samples were post-annealed at 400 °C for an hour under a 3 T magnetic field.

**Figure 2:**
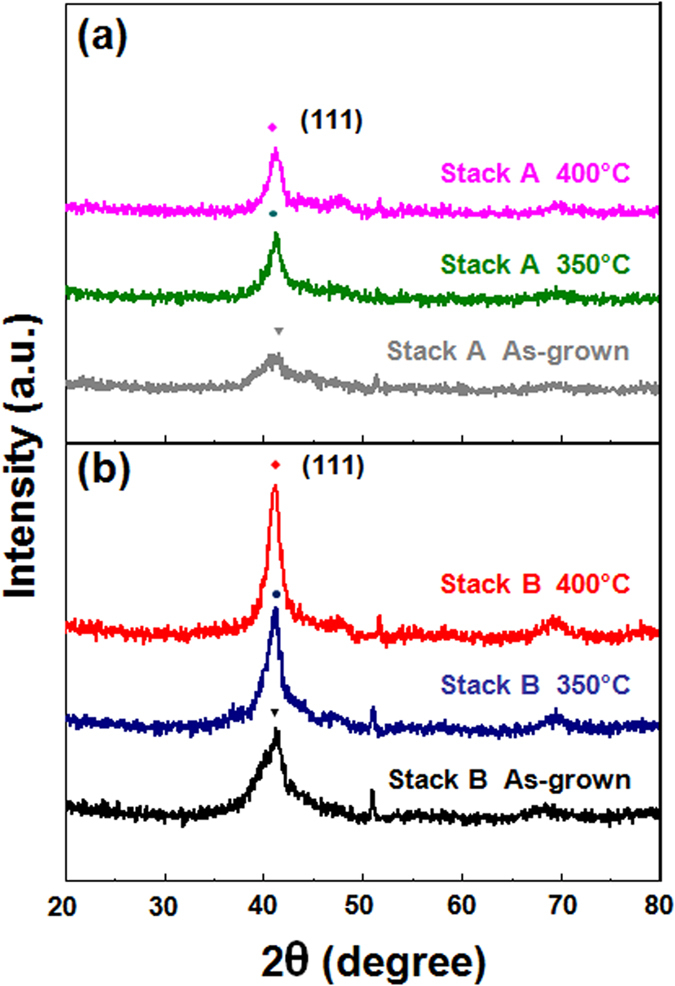
HR-XRD patterns of as-grown (grey, black lines), 350 °C post-annealed (blue, green lines) and 400 °C post-annealed (magenta, red lines) samples belonging to Stack A (**a**) and Stack B (**b**).

Two representative stacked samples were prepared as follows: Stack A: subs./Ta (3)/Ru (5)/Pd (3)/[Co (0.3)/Pd (0.3)]_3_/Co(0.5)/Pd (3) with a Co capping layer as a reference and Stack B: subs./Ta (3)/Ru (5)/Pd (3)/[Co (0.3)/Pd (0.3)]_3_/CoO(*t*_*CoO*_)/Pd (3) with a CoO capping layer. The numbers in the parentheses refer to the nominal thicknesses in nanometers and the subscripts for the Co/Pd multilayers refer to the stacking repetition numbers. Figure 1 presents the geometry of the stack architectures and representative magnetization curves for Stacks A and B annealed at 400 °C. As seen in Fig. 1(a), Stack A prepared without an oxygen insertion layer shows dominant in-plane magnetization with no PMA characteristics. More detailed observations of the as-grown, 350 °C, and 400 °C-annealed Stack A samples are shown in Supplementary Fig. S1, showing no clear PMA regardless of high-temperature annealing. However, as shown in Fig. 1(b), a Stack B sample with a 0.5-nm-thick CoO capping layer exhibits clear out-of-plane PMA hysteresis, reflecting the critical role of oxygen layer incorporation in PMA enhancement. Figure 2 plots HR-XRD *θ*–2*θ* diffraction patterns of Stack A and Stack B samples in the as-deposited and 350 °C and 400 °C annealed states. As seen in Fig. 2(a), Stack A samples show low intensity, broad peaks at around 41° regardless of annealing temperature. However, as shown in Fig. 2(b), as-grown and post-annealed Stack B samples show (111) orientation normal to the film surface in the 2*θ* region between 20° and 80°. Closer inspection of the as-grown Stack B samples reveals a narrower, more intense peak at around 41° than that of Stack A. With increasing annealing temperature, the (111) peak in the diffraction pattern of Stack B slightly shifts from 41.2° (as-grown) to 41.3° (350 °C and 400 °C), indicating lattice compression of the [Co/Pd] ML to match the atomic spacing in the Pd layer^15^. These XRD findings were consistent for all samples in our experiment upon annealing.

### CoO Capping layer oxygen content and thickness optimization, and magnetic characterization

**Figure 3:**
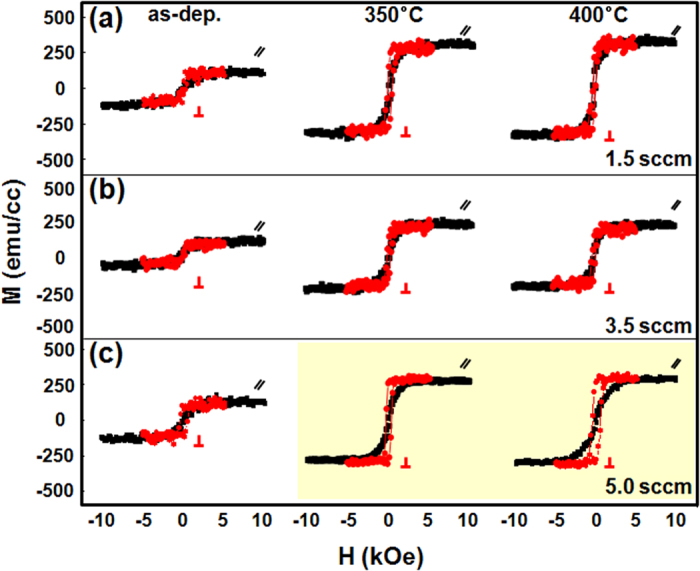
In-plane (black) and out-of-plane (red) magnetic hysteresis loops of as-grown, 350 °C, and 400 °C-annealed Stack B samples, where the CoO thickness was fixed at 0.5 nm and the oxygen content was varied by controlling the flow rate to (**a**) 1.5 sccm, (**b**) 3.5 sccm, and (**c**) 5 sccm.

**Figure 4:**
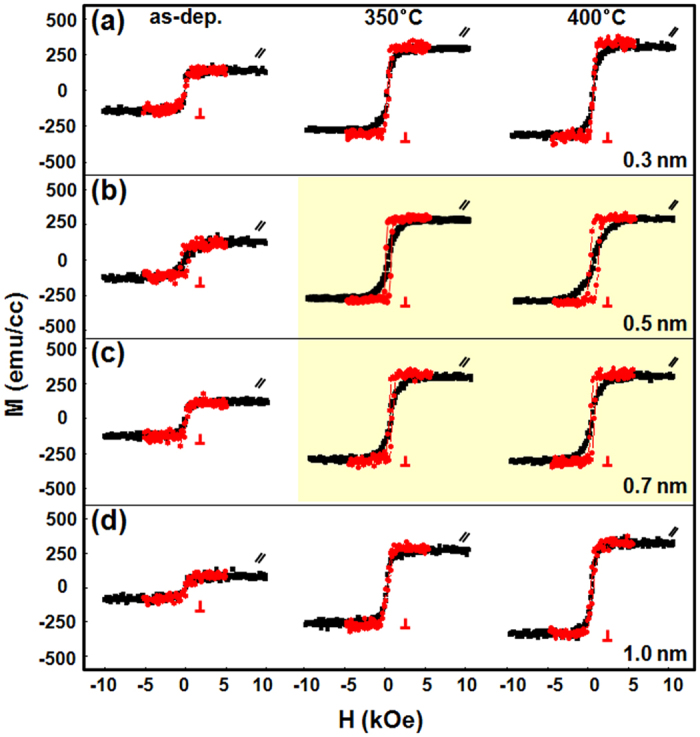
In-plane (black) and out-of-plane (red) magnetic hysteresis loops of as-grown, 350 °C, and 400 °C-annealed Stack B samples, where the fixed oxygen content was 5 sccm and the CoO thickness was (**a**) *t*_*CoO*_ = 0.3 nm, (**b**) *t*_*CoO*_ = 0.5 nm, (**c**) *t*_*CoO*_ = 0.7 nm, and (**d**) *t*_*CoO*_ = 1.0 nm.

To demonstrate the influence of the oxygen content in the 0.5-nm-thick CoO capping layer on the resulting PMA characteristics, in-plane and out-of-plane magnetic hysteresis loops of as-grown, 350 °C, and 400 °C-annealed Stack B samples are displayed in Fig. 3. In these samples the oxygen flow rates were set to 1.5, 3.5, and 5 sccm during growth of the CoO layer. As is evident in the figure, varying the oxygen content has a significant impact on the PMA enhancement after annealing. In particular, the sample with a 5 sccm oxygen flow rate (yellow background, Fig. 3c) exhibited enhanced PMA compared to the 1.5 and 3.5 sccm samples of Fig. 3(a,b), respectively. The in-plane (//) and out-of-plane (⊥) magnetic hysteresis loops of stack B samples in the as-deposited and 350/400 °C annealed states as a function of CoO capping layer thickness in the range of *t*_*CoO*_ = 0.3 nm to 1.0 nm are plotted in Fig. 4. As-grown samples exhibited no particular PMA regardless of the CoO thickness. Thus, a small number of MLs may be insufficient to sustain PMA at the interface between FM and NM layers due to the reduction of electron spin polarization. However, strong PMA appeared with 0.5 nm and 0.7 nm CoO layer thickness after proper heat treatment (yellow background) along with a distinct increase in *M*_*s*_. We suspect that the PMA enhancement upon annealing is due to atomic rearrangement or a phase change of the ultrathin ML matrix assisted by oxygen atom diffusion from the ultrathin CoO capping layer, as will be discussed later.

**Figure 5:**
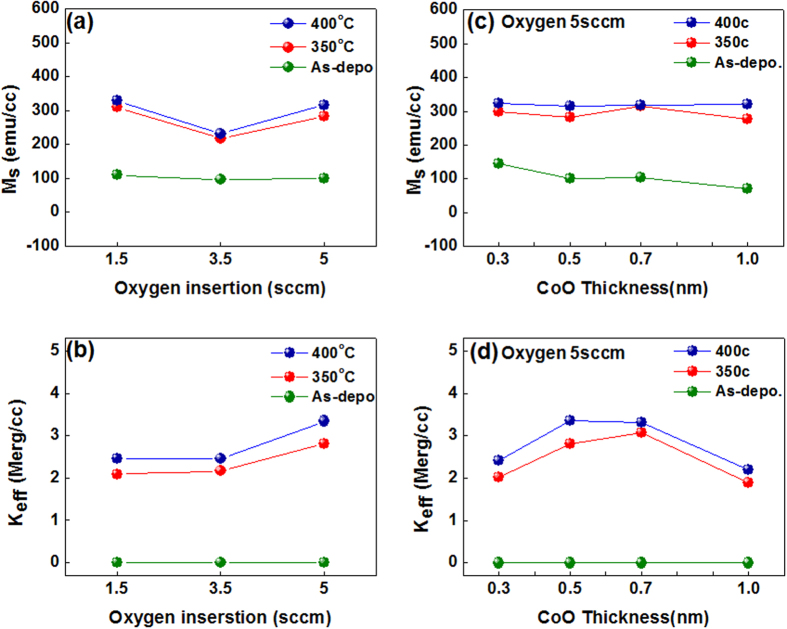
(**a**) Saturation magnetization (*M*_*s*_) per unit volume and (**b**) effective magnetic anisotropy energy (*K*_*eff*_) for as-grown (green line), 350 °C (red line), and 400 °C (blue line) post-annealed Stack B samples as a function of the oxygen content with a fixed CoO thickness of 0.5 nm. (**c**) Saturation magnetization (*M*_*s*_) per unit volume and (**d**) effective magnetic anisotropy energy (*K*_*eff*_) and for as-grown (green line), 350 °C (red line), and 400 °C (blue line) post-annealed Stack B samples as a function of CoO thickness.

Figure 5 shows the dependence of *M*_*s*_ on the oxygen incorporation per unit volume and the effective magnetic anisotropy (*K*_*eff*_) of stack B after 350 °C and 400 °C annealing. *K*_*eff*_ was calculated from the area enclosed between the in-plane and out-of-plane magnetic hysteresis loops using the well-known equation *K*_*eff*_ = *M*_*s*_ · *H*_*k*_/2, in which the *M*_*s*_ per unit volume is determined by dividing *m*_*s*_ by the [Co (0.3)/Pd (0.3)]_3_ volume, and *H*_*k*_ is defined by the large in-plane saturation field. Figure 5(a,b) present *M*_*s*_ and *K*_*eff*_ for Stack B as a function of the oxygen atom insertion with a fixed CoO layer thickness of 0.5 nm. As plotted in Fig. 5(b), the *K*_*eff*_ with 5 sccm oxygen flow is about 1.3 times higher than with 1.5 sccm and 3.5 sccm oxygen flow, implying that we can achieve better PMA characteristics by increasing the oxygen content in the film. As shown in Fig. 5(c), the *M*_*s*_ (red line) of the 350 °C-annealed sample increased dramatically, compared to the as-grown sample (green line). Furthermore, the *M*_*s*_ (blue line) of the 400 °C-annealed samples reached consistently higher values than the 350 °C-annealed samples at every thickness. The increase in *M*_*s*_ after high-temperature annealing is likely due to a phase transition and/or atomic structural realignment in the MLs. Figure 5(d) plots *K*_*eff*_ of 350 °C and 400 °C-annealed stack B samples, demonstrating enhanced PMA with increasing annealing temperature. The maximum *K*_*eff*_ value after 350 °C annealing was 2.81 (Merg/cc) at a 0.5 nm CoO thickness, and 3.07 (Merg/cc) at a 0.7 nm CoO thickness, which noticeably increased compared to *K*_*eff*_ value of 0.3 nm CoO thickness and 1.0 nm CoO thickness. The maximum *K*_*eff*_ value after 400 °C annealing further increased to 3.35 (Merg/cc) at a 0.5 nm CoO thickness, and 3.31 (Merg/cc) at a 0.7 nm CoO thickness. In addition, 425 °C annealing results in the slight decrease of maximum *K*_*eff*_ value, compared to those of 350 °C and 400 °C annealing as seen in Supplementary Fig. S2. These findings suggest that proper thermal treatment and a suitable choice of ultrathin capping layer can serve to enhance the PMA characteristics. To gain insight into the microstructure of Co/Pd MLs after high-temperature annealing, high-resolution transmission electron microscopy (TEM) of 350 °C-annealed samples from Stack B was conducted, along with Energy Dispersive X-ray Spectroscopy (EDS), as seen in Supplementary Fig. S3. TEM did not reveal obvious microstructural changes upon annealing, and the layer sequence as determined by EDS peak positions agrees with the order in which each element was deposited, recorded from the top side in the following order: Pd, Co, Ru, Ta, and Si.

### Structural analysis

**Figure 6:**
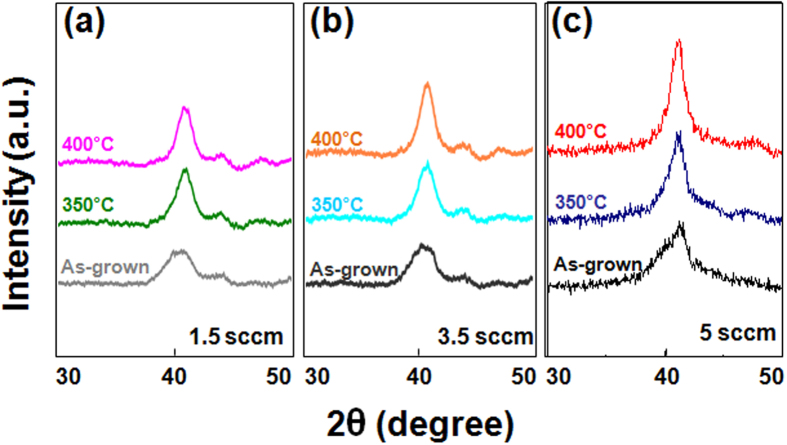
HR-XRD patterns of as-grown, 350 °C and 400 °C post-annealed Stack B samples as a function of oxygen content with a fixed CoO thickness of 0.5 nm: (**a**) 1.5 sccm, (**b**) 3.5 sccm, and (**c**) 5 sccm.

To verify the effect of oxygen atom insertion, HR-XRD patterns of as-grown, 350 °C, and 400 °C post-annealed Stack B samples were recorded as a function of the oxygen content, in which the oxygen flow was varied in the range of 1.5 sccm to 5 sccm during growth, with a fixed CoO layer thickness of 0.5 nm. As seen in Fig. 6, the 5 sccm sample exhibited stronger peaks than the 1.5 sccm or 3.5 sccm samples. In addition, all samples exhibit slight peak shifts to higher angles after 350 °C and 400 °C annealing; for example, the 1.5 sccm and 3.5 sccm samples show slight peak shifts from 40.5° to 40.8°, and from 40.2° to 40.8°, respectively. The 5 sccm sample also shifts to higher angle upon annealing. The similar degree of peak shifting in all samples suggests that the origin of the shifting is the same, possibly reflecting lattice contraction to match the [Co/Pd] ML atomic spacing during annealing. The enhancement of the peak intensity of the 5 sccm sample seems to depend on the oxygen content inside the CoO capping layer, possibly contributing to better crystal orientation and/or lattice strain to match the Pd layer atomic spacing upon annealing^15^. However, further experiments are needed to establish a clearer explanation for the enhancement observed in the 5 sccm sample.

**Figure 7:**
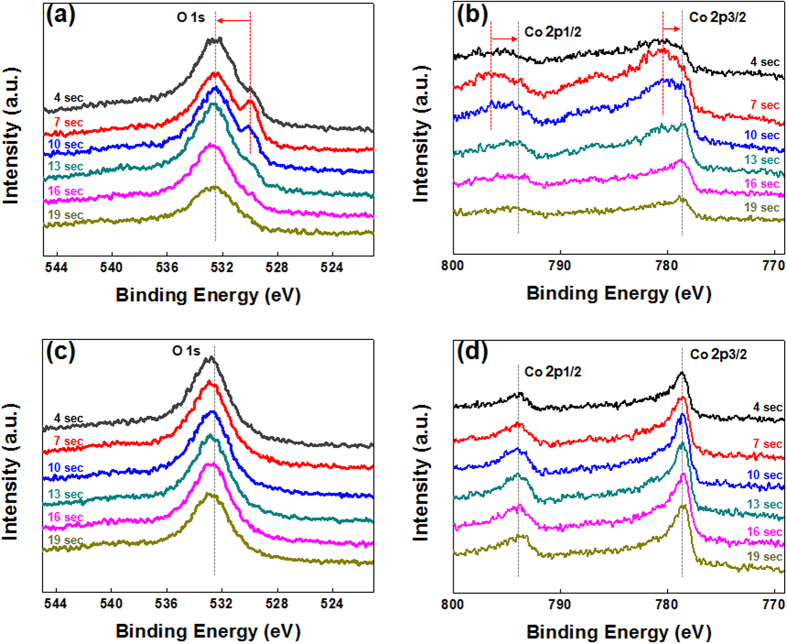
XPS spectra of as-grown and annealed Stack B samples as a function of etching time ranging from 4 s to 19 s. (**a**) O 1 s and (**b**) Co 2p XPS spectra for as-grown samples. (**c**) O 1 s and (**d**) Co 2p spectra for 350 °C post-annealed samples.

To clarify the role of the ultrathin CoO capping layer in the enhanced PMA characteristics, XPS depth profiles were taken for as-deposited and 350 °C-annealed Stack B samples using Al *Kα* radiation (1486.6 eV) with a spot size of 400 μm. All spectra were measured in the constant analyzer energy mode with a pass energy of 100 eV. Etching was carefully controlled in 3 s time steps for 30 total steps until substrate spectra were visible. The C 1 s binding energy (284.4 eV) was used as an energy reference for the calibration of all XPS spectra. Spectra are compared at 4, 7, 10, 13, 16, and 19 s etching times. O 1 s and Co 2p peaks of the as-grown samples before etching are plotted in Fig. 7(a,b), respectively. As seen in Fig. 7(a), two O 1 s components are identified at 532.38 eV and 530.03 eV. The higher binding energy peak at 532.38 eV is most likely due to weakly bonded oxygen species, such as CO_3_, OH, or adsorbed O_2_ on the film’s surface^16^, while the lower binding energy peak is attributed to Co-oxygen bonding in the CoO capping layer^17^. The low binding energy peaks were present up to 13 s of etching time, and represent oxidized Co-O phases initially created by the ultrathin CoO capping layer. After etching for 13 s these low binding energy peaks disappeared, indicating the absence of metal-oxygen bonding. Figure 7(b) plots the two main Co 2p_1/2_ and 2p_3/2_ peaks of as-grown Stack B at 796.78 eV and 780.88 eV, respectively. These peaks are associated with the presence of mixed phases such as CoO, Co_2_O_3_, or Co_3_O_4_ in the as-grown state^18^. With increasing etching times, the main Co 2p_1/2_ and 2p_3/2_ peaks shift to lower binding energies up to 13 s, and then two peaks appear at 793.03 eV and 778.68 eV corresponding to metallic Co bonding in the bottom region of the [Co/Pd]_3_ ML matrix. These observations confirm the presence of an oxidized phase induced by the ultrathin CoO_x_ capping layer in the [Co/Pd]_3_ MLs at etching times ranging from 7 s to 13 s. In addition, the initial peaks before 13 s had relatively broad widths and low intensities compared to peaks after 16 s and 19 s. Therefore we believe that the broad width and smooth peak shifting indicate the formation of several Co-O sub-oxides in the as-grown state. Figure 7(c) shows the O 1 s peaks of 350 °C annealed Stack B samples showing the clear absence of the Co-O peak (530.03 eV) previously observed in the as-grown state, with only a sharp metallic peak (532.38 eV) remaining. Figure 5(d) also shows an absence of Co-O peaks (796.78 eV and 780.88 eV) after 350 °C annealing. Thus, we infer that high-temperature annealing breaks the initially weak Co-O bonds in the top region of the ML matrix, leading to structural realignment through the diffusion of thermally activated oxygen atoms.

### Possible geometry configuration

**Figure 8:**
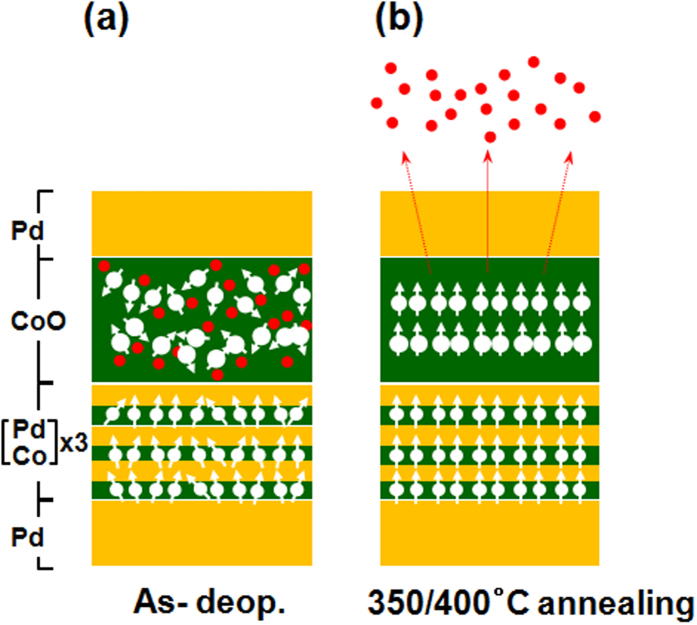
Possible geometry configurations for (**a**) as-grown and (**b**) annealed stack B samples, where temperature-dependent diffusion of oxygen atoms or ions may induce possible structural reconfiguration in the ML matrix.

Based on these observations, we propose a physical model to elucidate magnetic and structural behaviors of the as-grown and annealed Stack B samples, illustrated in Fig. 8. Initially, the XPS data of Fig. 7 support the possible coexistence of various CoO, Co_2_O_3_, Co_3_O_4_, or metallic Co phases in the as-grown CoO capping layer prepared during RT sputtering. Therefore, the ultrathin CoO layer will initially have paramagnetic or amorphous features, not antiferromagnetic (AF) characteristics. Furthermore, the as-grown CoO capping layer is likely to have weak oxygen bonding states, as shown in Fig. 8(a). Thus, annealing at high temperature may break the weak Co-O bonds, allowing thermally activated oxygen atoms or ions to diffuse into the MLs. Then, diffusing oxygen atoms may induce enough lattice strain to cause atomic reconstruction in the [Co/Pd] MLs, contributing to an enhancement in the PMA, as displayed in Fig. 8(b). However, more detailed studies are required to clarify the structural characteristics in ultrathin MLs containing an ultrathin oxide layer and confirm our model.

## Discussion

In this work, we report PMA in ultrathin [Co/Pd]_3_ MLs upon incorporation of a CoO capping layer after high-temperature annealing. Control of the capping layer thickness, post-annealing conditions, and oxygen content is critical for achieving enhanced PMA. Structural investigations provide evidence for efficient diffusion of thermally activated oxygen atoms associated with initially weak Co-O bonds created during the RT preparation of CoO_x_ layer. Thus, enhancement of the PMA may be ascribed to structural reconfiguration due to lattice strain relaxation in an ultrathin [Co/Pd] ML framework upon high temperature treatment. We anticipate that the ability to enhance PMA characteristics via incorporation of a suitable oxide capping layer may eventually meet the demand for thermally stable PMA spintronic devices.

## Methods

Two representative stacked samples were prepared on substrate/Ta (3)/Ru (5)/Pd (3) seed layers at room temperature (RT) using dual DC & RF magnetron sputtering systems. (Numbers in parentheses refer to the thickness of the layer in nanometers.) The stack sequence is as follows. Stack A: subs/Ta (3)/Ru (5)/Pd (3)/[Co (0.3)/Pd (0.3)]_3_/Co(*0.5*)/Pd (3) with a Co capping layer as a reference and Stack B: subs./Ta (3)/Ru (5)/Pd (3)/[Co (0.3)/Pd (0.3)]_3_/CoO(*t*_*CoO*_)/Pd (3) with a CoO capping layer. The base pressure of the sputtering system was less than 5 × 10^−8^ Torr, and the working pressure was set to 3 × 10^−3^ Torr during sample growth. The CoO layer serving as a capping layer was sputtered using a pure Co target in an Ar (25 sccm)/O_2_ (5 sccm) atmosphere, and the thickness *t*_*CoO*_ was varied from 0.3 nm to 1.0 nm. The Co/Pd ML was deposited by alternately sputtering Co and Pd targets, and every sample was post-annealed at temperatures ranging from 350 °C to 400 °C for 1 hour in vacuum (less than 10^−6^ Torr) in a 3 T perpendicular magnetic field. In order to determine the role of oxygen inside the CoO capping layer, three samples with different oxygen content (controlled by varying the flow rate to 1.5, 3.5, and 5 sccm) were prepared, with fixed CoO thickness (*t*_*CoO*_ = 0.5 nm).

## Additional Information

**How to cite this article**: Chung, W. S. *et al*. Ultrathin Co-O oxide layer-driven perpendicular magnetic anisotropy in a CoO/[Co/Pd]_m_ multilayer matrix upon annealing. *Sci. Rep.* **6**, 37503; doi: 10.1038/srep37503 (2016).

**Publisher’s note:** Springer Nature remains neutral with regard to jurisdictional claims in published maps and institutional affiliations.

## Supplementary Material

Supplementary Information
