# Supplementary material for: Ultrathin Co-O oxide layer-driven perpendicular magnetic anisotropy in a CoO/[Co/Pd]m multilayer matrix upon annealing
Source: Sci Rep. 2016 Nov 25;6:37503. doi: 10.1038/srep37503 (PMC5122862; doi:10.1038/srep37503)
Supplement: Supplementary Information [file srep37503-s1.pdf]

Ultrathin Co-O oxide layer-driven perpendicular magnetic anisotropy in a $CoO/[Co/Pd]_m$ multilayer matrix upon annealing

Woo Seong Chung,[1] Seung Mo Yang[2], Tae Whan Kim,[1] and Jin Pyo Hong[2,]*

[1]*Nano Quantum Electronics Lab, Department of Electronics and Computer Engineering, Hanyang University, Seoul 133-791, South Korea*

[2]*Novel Functional Materials and Devices Laboratory, Research Institute for Natural Science, Department of Physics, Hanyang University, Seoul 133-791, South Korea*

Supplementary Information

1. Magnetic hysteresis loops of the as-grown and annealed Co capping without oxygen insertion (Stack A)

As shown in Fig. S1, Stack A with a Co capping layer containing no oxygen exhibited no clear PMA regardless of annealing, in which in-plane dominant magnetization was obtained without particular change.

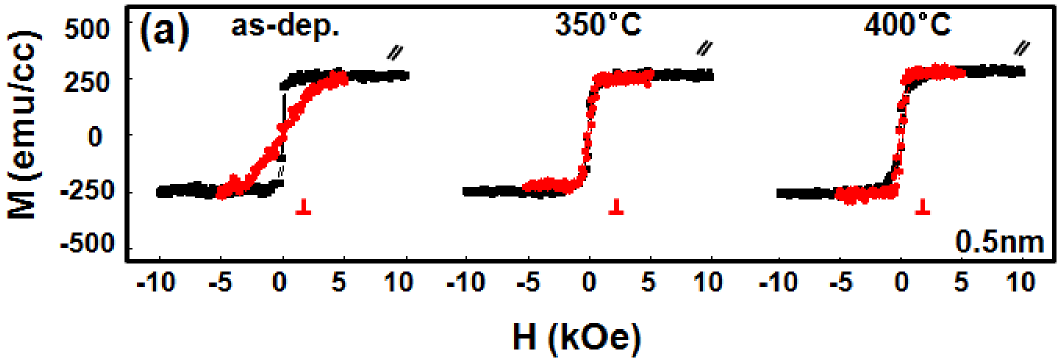

Fig. S1. Magnetic hysteresis loops of as-grown, 350℃, and 400℃-annealed Stack A, where the Co capping thickness was t_{Co}= 0.5 nm

2. Magnetic hysteresis loops and K_{eff} value of 425°C-annealed Stack B

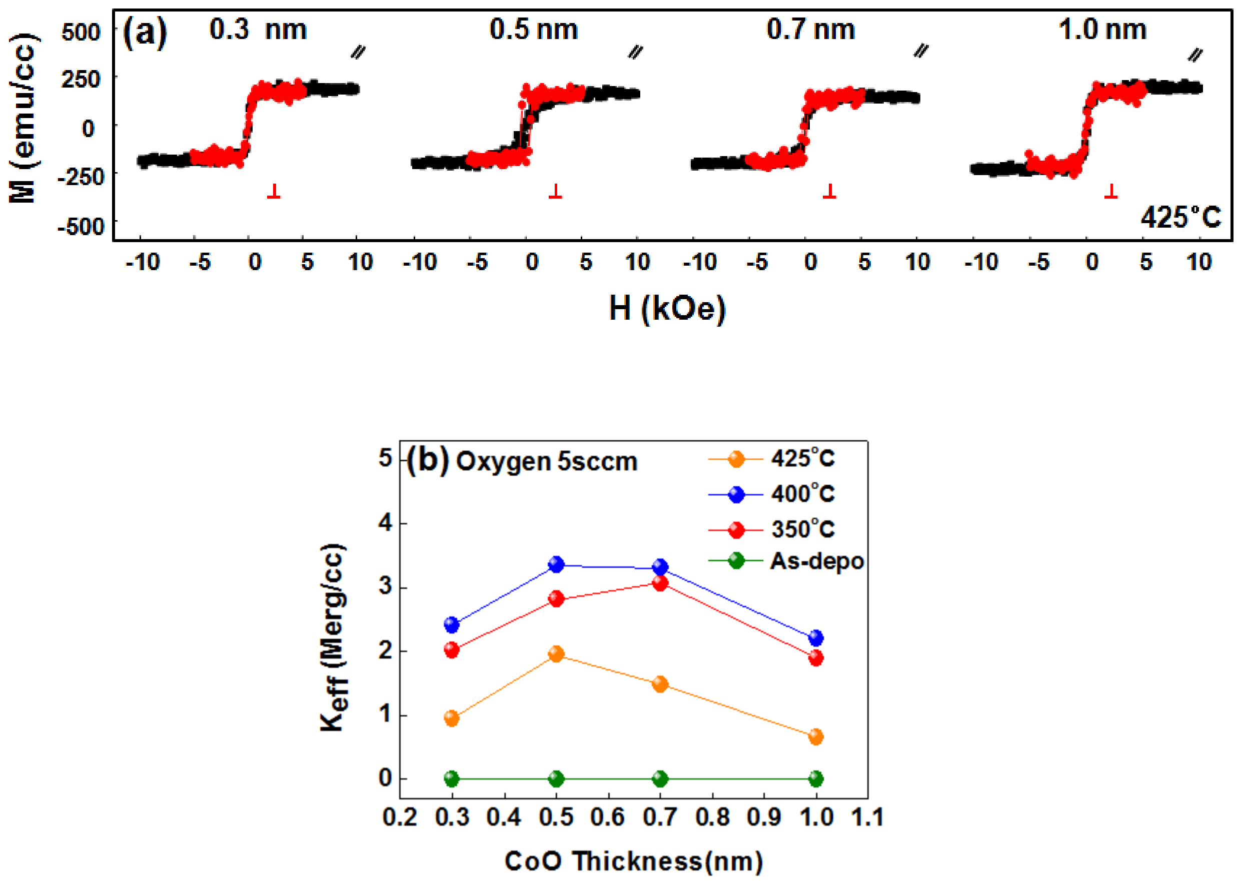

Fig. S2. (a) Magnetic hysteresis loops of 425℃-annealed Stack B, where the Co capping thickness was t_{CoO}= 0.3 ~ 1.0 nm, (b) effective magnetic anisotropy energy (K_{eff}) for as-grown (green line), 350℃ (red line), 400℃ (blue line), and 425℃ (orange line) post-annealed Stack B samples as a function of CoO thickness.

3. TEM images and corresponding EDS analyses of 350°C-annealed Stack B

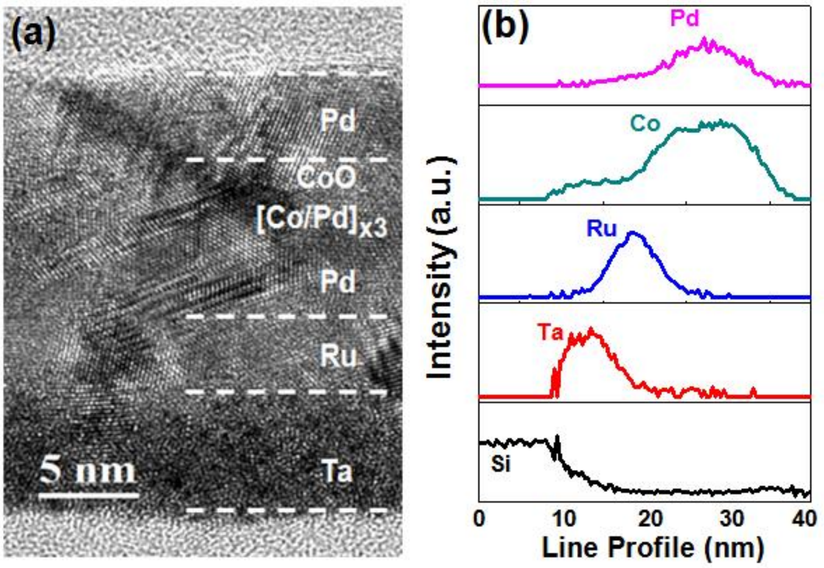

Fig. S3. (a) TEM images and (b) corresponding EDS analyses of 350℃-annealed Stack B, in which oxygen was not detected due to the extremely small amount of oxygen inside the CoO capping layer and significant out-diffusion during annealing.
